# Integrating Trap-Neuter-Return Campaigns Into a Social Framework: Developing Long-Term Positive Behavior Change Toward Unowned Cats in Urban Areas

**DOI:** 10.3389/fvets.2018.00258

**Published:** 2018-10-24

**Authors:** Jennifer L. McDonald, Mark J. Farnworth, Jane Clements

**Affiliations:** ^1^Cats Protection, National Cat Centre, Haywards Heath, United Kingdom; ^2^Bristol Veterinary School, University of Bristol, Bristol, United Kingdom; ^3^Animal, Rural and Environmental Sciences, Nottingham Trent University, Southwell, United Kingdom

**Keywords:** Domestic cat, *Felis catus*, urban environment, behavior change, neuter, TNR, stray cat, unowned cat

## Abstract

Cat management is often discussed in terms of population reduction, with trap-neuter-return (TNR) campaigns commonly organized to manage unowned urban cat populations. However, long-term effectiveness is only possible if positive neutering practices are continued by local residents. Here we discuss how implementing TNR within a wider framework of social engagement has the potential to tackle cat overpopulation and instill long-term positive behavior change toward them. We demonstrate how community engagement pre-TNR can help establish a baseline of the attitudes, knowledge and behavior concerning cats. Using a case study, we explore whether this information can be linked with positive intended behavior based on intentions to arrange for neutering of unowned cats. Structural equation modeling indicated that negative attitudes toward cats and reduced knowledge around neutering reduced the likelihood of positive intended behavior. This result was underpinned by the indirect effects of perceptions of unowned cats and reduced understanding of their needs. Utilizing these results alongside an understanding of the values and motivation of the community allows for tailored and targeted education and intervention. In turn, this addresses the underlying knowledge gaps and perceptions regarding cat welfare. This framework can help address the challenge of cat management because it: (1) takes an integrative approach to identifying the motivations of communities to take responsibility for unowned cats; (2) changes the structure of the social environment, encouraging positive neutering practices for unowned cats. In turn this improves the impact and longevity of TNR campaigns whilst promoting positive welfare change for unowned and owned cats; and (3) appreciates that opinions are likely to vary hugely between areas, therefore providing an adaptable community level approach.

## Introduction

Human behavior change is fundamental to tackling anthropogenic problems, both globally and locally. The unowned cat overpopulation problem in urban areas is one such issue, largely a function of socio-demographic parameters (1–3), human behavior (4), and attitudes (5, 6). Large numbers of free-roaming unowned cats can be found in areas of high human population density where they are an integral, if sometimes contentious, part of the community. Although no accurate population estimates for unowned cats exist in the United Kingdom (UK) their prevalence in urban areas is often of animal-welfare (7–9), public-health (8, 10), and environmental (9) concern. Human behavior is a key contributor to unowned cat populations, with abandonment of unneutered cats and unwanted litters providing a persistent source of unwanted cats in the environment (4). Additionally, the provision of neutering for unowned cats will largely be influenced by a community's capability, opportunity and motivation to care for unowned cats. Consequently, the long-term impact of any management that aims to control breeding, such as trap-neuter-return (TNR) campaigns, can be undermined by local neutering practices, with TNR treating the symptoms of the overpopulation problem but not necessarily the cause.

The dynamic nature of unowned cat populations, influenced by constant immigration, and emigration (4), necessitates intensive and persistent neutering campaigns of unowned cats to prevent increases in population size (11–13). Such TNR work is both time and resource intensive, yet is at risk of becoming insignificant in the long-term if human behaviors and attitudes within the community are not taken into account (3, 4). The barriers which exist for people to conduct their everyday lives can impact hugely on any other desirable behavior toward cats (3), especially as the unowned cat population is more likely to be a problem in highly deprived areas (1–3). Consequently, barriers and motivators for different behaviors toward cats will vary between people and across communities, being somewhat reflective of cultural, social, or economic differences (3). Therefore, untargeted interventions alone are likely to receive modest and variable success without looking at behaviors in the community.

Assuming the provision of affordable neutering services, the capacity of local people to arrange neutering for their cat and/or for unowned cats in their community, will still be strongly influenced by their perceptions and beliefs. Some studies have shown that the intention to neuter unowned cats is predicted by religious beliefs, attitudes toward neutering and beliefs about personal capacity (6). Additionally, the provision of care for unowned cats is influenced by feelings toward them (14). Understanding these psychological factors will allow community awareness campaigns and interventions to approach the issue of neutering in a way that is consistent with cultural, social and economic circumstances.

Here we propose a modeling framework to identify key factors underpinning positive neutering behavior in a community. We illustrate the potential for TNR campaigns to not only have short-term operational benefit but, through community engagement and behavior change interventions, also the potential to empower a community to ensure the continuation of positive neutering practices for the cat population as a whole. We provide an example of how a modeling approach can unravel the beliefs underpinning a positive intended behavior, and how this could potentially be used for further community engagement.

## Development of a behavior change framework

### Behavioral conditions

Education often forms the focus of any campaign to bring about behavior change, whether based on health, conservation or animal welfare. A plethora of models exist that set out to provide a deeper understanding of the psychological processes underpinning behavioral change. However, it is increasingly recognized that knowledge alone is insufficient, because many other factors influence behavior change including self-efficacy (15), social norms (16, 17) and habits (18) to name but a few. More recently these wide ranging behavioral conditions have been grouped within a single tool, the behavior change wheel (19). For the purposes of this study we will discuss behavioral concepts using the behavior change wheel, but we recognize that this framework is a synthesis of pre-existing frameworks.

Individual behavior can be driven by three essential components; capability, opportunity and motivation, termed the COM-B model (Table 1). Capability is an individual's ability to engage in a behavior including physical and psychological barriers to performance. Opportunity considers external factors that prompt or enable the individual to perform the target behavior. These include social opportunity based on the positive or negative influences of social norms and community values and physical opportunity determined by situational or environmental factors. Motivation includes all internal factors that trigger behavior, including knowledge-based, reflective, and conscious motivation and automatic impulsive and emotionally driven motivation. Effective behavior change therefore requires maximizing capability to regulate one's own behavior, maximizing opportunity to support desired behavior, increasing motivation to engage in desired behavior and reducing motivation to continue with undesired behaviors. Understanding these key principles of behavior change allows development of tailored interventions.

**Table 1:** COM-B factors, interventions and behavior change techniques in relation to the behavior of reporting stray cats for neutering.

**COM-B categories**	**Definition**	**Relevance of COM-B component**	**Intervention function(s)**	**Example behavior change technique**
Capability-physical	Capacity to physically engage in the behavior	N/A—People would generally have the physical ability to report stray cats		
Capability- psychological	Capacity to engage in the thought processes that underpin the behavior	Lack of knowledge about who to report to and how	Training	Demonstration of the behavior and instruction on how to perform the behavior
Opportunity-social	The social and/or cultural features that enable a behavior	No support or prominent community role models obviously doing the behavior	Environmental restructuring; Modeling; Enablement	Restructuring of social environment, providing social support and demonstration of behavior
Opportunity-physical	Situational or environmental features that enable the behavior	No resources or opportunities provided	Training; Environmental restructuring; Enablement	Restructuring the social environment by providing routes to report unowned cats
Motivation-reflective	Conscious thought processes	Worries about what to do, how to report pets, lack of knowledge and confusion	Enablement	Restructuring social environment and providing social support e.g. providing tools to enable the intended behavior
Motivation-automatic	Automatic thought processes driven by impulses, emotions and beliefs	Reporting unowned cats not habitual behavior	Training; Environmental restructuring; Enablement	Restructuring social environment, providing social support, and demonstration of the behavior

There are nine intervention functions: Education, Persuasion, Incentivisation, Coercion, Training, Enablement, Modeling, Environmental Restructuring, and Restriction [see (19) for a full summary]. A key first step to deciding interventions is to define the problem and understand the barriers to, and facilitators of, positive change.

### Applying a behavioral change framework to TNR

Local neutering practices of both owned and unowned cats will be important factors in driving the number of unowned cats in the community. Therefore, desired behaviors include arranging or taking unowned cats to the veterinarians to be neutered and early neutering of owned pets. Barriers to positive neutering practice may be due to limited capability, motivation and/or opportunity (examples in Table 1). Consequently, it is essential to engage with communities to understand specific and localized drivers of, and barriers to, desirable behavior toward cats.

Applying a systematic method for selecting behavior change techniques includes, in the first instance, making a behavioral diagnosis and identifying which of the behavioral conditions are important barriers. This is then linked to specific interventions that, in turn, can guide the most relevant behavior change techniques (Table 1). By undertaking this action, TNR interventions may be in a much stronger position to have a long-term and perpetuated impact in local communities.

In practice, understanding and modeling such a complex system firstly requires engaging with the community and using a survey-based approach and/or detailed focus groups to understand behaviors of interest and their potential underpinnings. Secondly, it requires an adaptable modeling framework that is capable of integrating a comprehensive set of behavioral concepts. The framework should allow for robust hypothesis testing and development of theories regarding the ways people think about cats.

### Engagement and targeted campaigns

Once the behaviors of interest have been identified, data concerning those behaviors should be gathered at the start of the campaign. Surveys and/or focus groups can be used as tools to explore the range of different behavioral barriers previously described. Surveys and community engagement prior to TNR has three direct benefits; (1) data can be gathered to explore barriers to positive neutering practices (2) areas of high unowned cat density can be identified in advance via community knowledge, and (3) buy-in and awareness of TNR within the community is increased prior to implementation.

Data gathering methods will be resource and area dependent. Although in-depth discussion of survey implementation is beyond the scope of this article, generally face-to-face surveys are likely to deliver the most representative results, yet are also the most expensive, requiring the use of highly trained interviewers. Telephone or postal surveys may provide a good alternative. Online surveys are often used as a cost-effective means to gather data, however they are seldom representative of the general population, due to biases related to internet use and access. With all approaches, careful consideration of biases will need to take place and weighting should occur if samples are not representative (20). Questions should target the behavior of interest, behavioral categories and demographic information to ensure representative responses. Additionally, engagement within the community provides an opportunity to identify areas where TNR should be focussed by asking about the number and location of unowned cats in the area.

The knowledge from focus groups and/or surveys can be used to improve community understanding around cats. It may also involve them in solutions that are acceptable whilst tailoring culturally appropriate information to empower individuals within communities. Such processes may function to embed positive behavioral changes regarding cats which persist into the future. Interventions such as posters, social media, leaflets, public events, school visits, and local TV and radio can all be used to ensure the community remains at the heart of the campaign.

### How to analyse survey data

With results of large surveys, an exploratory phase is often helpful in order to evaluate the key relationships between variables. Principal component analysis (PCA) distills multiple correlated variables into singular axes and indicates the degree to which survey items load onto those axes. It is a useful tool to reduce the dimensions of the data, condensing large datasets based on the correlations among multiple survey questions. This is often a helpful first step to understand the underlying correlations that account for most of the variation and structure the data.

Utilizing results from exploratory PCA analyses, structural equation modeling (SEM) can provide a more process-led approach, considering survey items as part of a system with inter-dependent relationships, both correlative and causative. It does so by incorporating a network of equations that accounts for composites of variables, which underlie latent social constructs. For example, perceptions of unowned cats may be underpinned by several survey questions, including both negative and positive perceptions. In this scenario, perceptions would be a latent (unobserved) variable. Multiple latent variables, determined by correlated survey variables, can be incorporated in the model. This allows for simultaneous assessment of interrelationships among different social constructs, whilst including several independent and dependent variables e.g. perceptions, attitudes, knowledge, intended behavior and compliance behavior. The first step of SEM therefore focuses on creating the latent constructs that comprise the various elements of the framework. SEM then provides a means to assess how those constructs are related and the directionality of significant relationships, offering a straightforward method of addressing multiple relationships simultaneously. Consequently, this allows for the testing of theoretical psychological frameworks. SEMs are particularly well suited to model multiple associations within a survey. They combine correlated variables and apply multivariate techniques to determine how interacting concepts influences a key question (or latent variable) of interest. SEMs can therefore, be applied to explore drivers of intended or reported positive behaviors toward cats, assessing which social constructs underpin those behaviors.

## Case study

Using a case study, we provide an example of how TNR campaigns can be part of a wider community engagement program to initiate positive behavior change toward cats.

### Study area

Bulwell is an old English market town of ~8,000 households about 4.5 miles northwest of Nottingham, England. It was chosen as an area where unowned cats were thought to be prevalent from previous charity work in the community. Additionally, Bulwell was in the 10% most deprived wards in the UK (21), therefore perhaps more likely to have cat overpopulation (2) and animal welfare issues.

### Engagement

**Figure 1:**
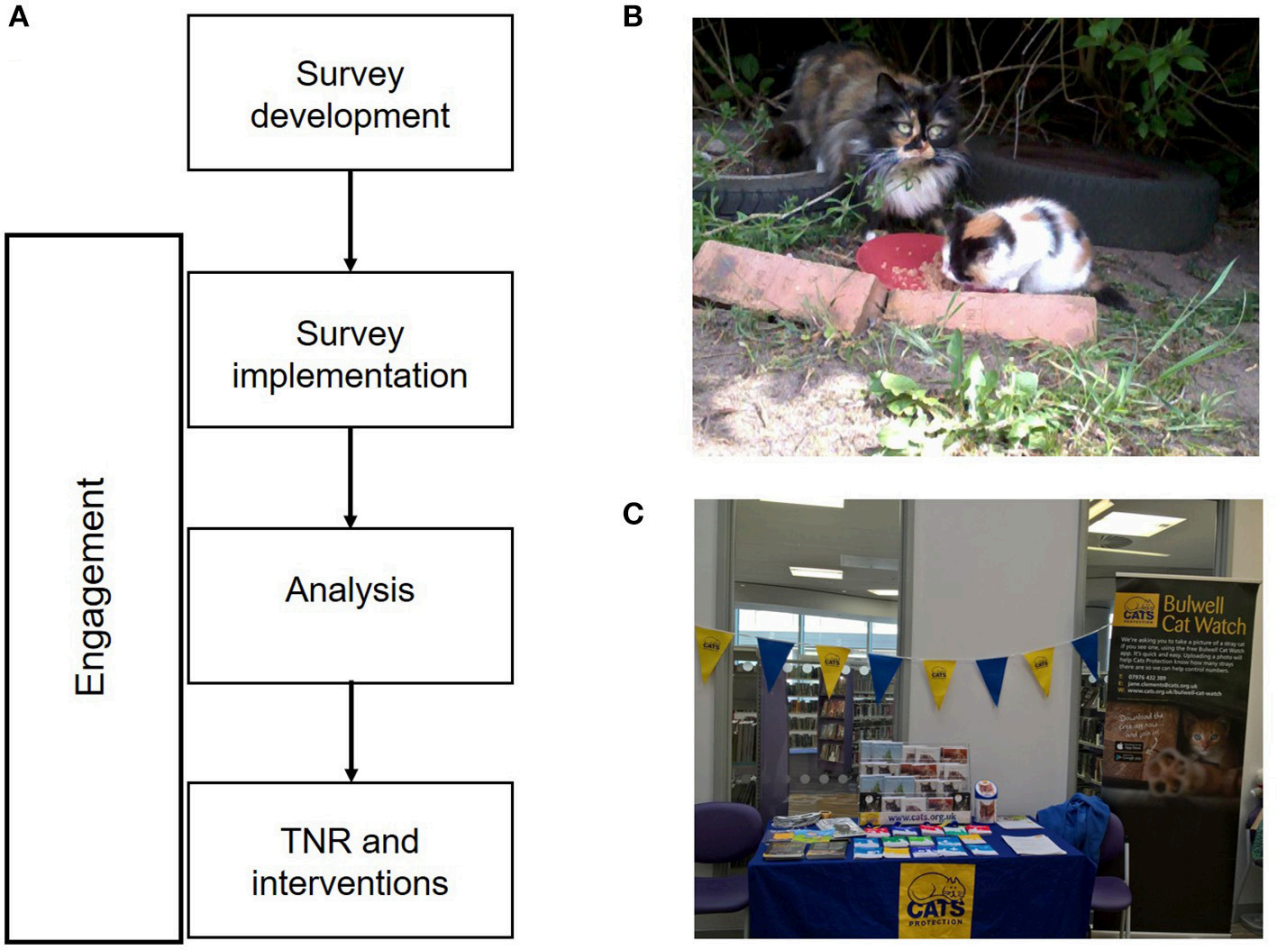
**(A)** Framework for engagement and TNR activities. **(B)** Stray cats were reported by local residents **(C)** Example of wider community engagement program.

Engagement started in September 2016, before the commencement of the TNR operations, and continued throughout the campaign (Figure 1). It consisted of a variety of communication methods including a combination of face-face engagement and one-way communications. The campaign was called “Bulwell Cat Watch,” to maintain a sense of community ownership to the project.

Face-to-face engagement included interviews with residents. To build local awareness of, and trust in, the team a drop-in point was also established. This provided a social hub for interested residents to talk to the outreach team, report on unowned cats and find out more about the work within the community. To target a wider audience numerous community events were held such as fun days alongside team attendance at other local community events. Attending and holding events reached out to people who would not otherwise proactively engage, yielding new information and engaging new audiences (Figure 1).

Information regarding areas where unowned cats are reported to occur (cat hotspots) were used to target leafletting prior to TNR. Posters and leaflets were also provided in local shops and targeted Facebook adverts were used for online communications.

In addition, a Facebook group was set up for the project that was largely community led. Content and conversations were decided by the participating Bulwell residents, with the community outreach team contributing and responding to posts. This online community shared images or reported sightings of unowned cats, reported lost, or found pet cats and shared advice around looking after local cats. The group engaged cat-lovers, the target audience for taking action, encouraging reporting of and responsibility for unowned cats. The nature of Facebook groups means that their membership and content builds and evolves organically, providing a low-resource way to reach out to and engage with residents.

Offering different channels for people to report cats maximizes participation. A mobile application was developed to provide another means to support residents to report cats easily and accurately. Local news coverage also introduced Cat Watch to residents and provided progress updates.

### Survey

#### Overview

A cross-sectional random-sample survey was carried out with residents. Field researchers from The Campaign Company (TCC) went door to door to conduct face-to-face interviews over 3 weeks in July 2016.

Survey questions were designed to assess the likelihood of individuals taking or arranging for neutering of unowned cats, as a measure of positive intended behavior. Behavioral intention is thought to directly influence behavior (22). To understand what drives such intentions a range of questions were asked around awareness and knowledge of cat welfare and neutering, barriers, and motivators for neutering cats and socio-demographic status. Additional questions were asked about the number of unowned cats in local areas, providing an indication of their locations and therefore, operational value to identify areas where both TNR and engagement may be most beneficial. In total twenty questions were asked around unowned and owned cats in the community (see Supplementary Table 1). Additional questions concerning the demographics and profiles of respondents were also included, but are beyond the scope of this study and not discussed further here. The survey took on average 15 min to complete.

Respondents totalled 776 of which, 23% (*n* = 178) owned a cat and 49% (*n* = 377) stated they liked cats either a little or a lot. However, most people (87%) identified negative consequences of unowned cats in the community, with dirtiness and smell the most commonly stated reasons, followed by fighting with pet cats and noise (Table 2). Unowned cats breeding with pet cats was identified as a problem by a small minority (14%) of respondents (Table 2).

**Table 2:** Percentage distribution alongside sample size of respondents' responses to key survey questions.

	**% (*n*)**
**PERCEPTIONS OF UNOWNED CATS**	
Think that there are negative consequences of unowned cats in the community	87 (676)
Cite the following bad points of unowned cats include:	
Dirty	40 (309)
Smell	20 (157)
Fighting with pet cats	18 (137)
Noise	17 (130)
Breeding	14 (112)
Think that there are positive consequences of unowned cats in the community	42 (321)
Good because they control vermin	20 (156)
**KNOWLEDGE OF UNOWNED CATS NEEDS**	
Think that it is very or quite important that unowned cats are provided with neutering	74 (571)
Think that it is very or quite important that unowned cats are provided with treatment	72 (559)
Think that everyone in the community are responsible for looking after unowned cats	18 (137)
Think that charities are responsible for looking after unowned cats	39 (302)
**KNOWLEDGE OF NEUTERING**	
Disagree that related cats won't mate with each other	37 (290)
Agree that neutering reduces anti-social cat behavior, like wailing and spraying	67 (519)
Disagree that female cats should be allowed to have kittens before being neutered	47 (364)
**BEHAVIORAL INTENT**	
Likely to arrange or take an unneutered cat, which you believe to be unowned, to the vet to be neutered	25 (192)

Knowledge of neutering showed substantial variation. Only 37% of people correctly disagreed with the statement that “related cats would not mate with each other,” with the remainder either agreeing or unsure. Over two-thirds of respondents agreed that neutering reduces antisocial cat behavior such as wailing and spraying, with the remainder either disagreeing or not sure (Table 2).

The majority of respondents thought it was very or quite important to provide neutering and veterinary treatment for unowned cats. However, only 18% of people thought that the community were responsible for looking after unowned cats, with charities considered responsible more often (39%; Table 2).

The majority of people (68%) said they were unlikely or very unlikely to arrange or take an unowned cat to be neutered, with only 25% stating they were likely to engage with unowned cat neutering (Table 2).

#### Model outcomes

**Figure 2:**
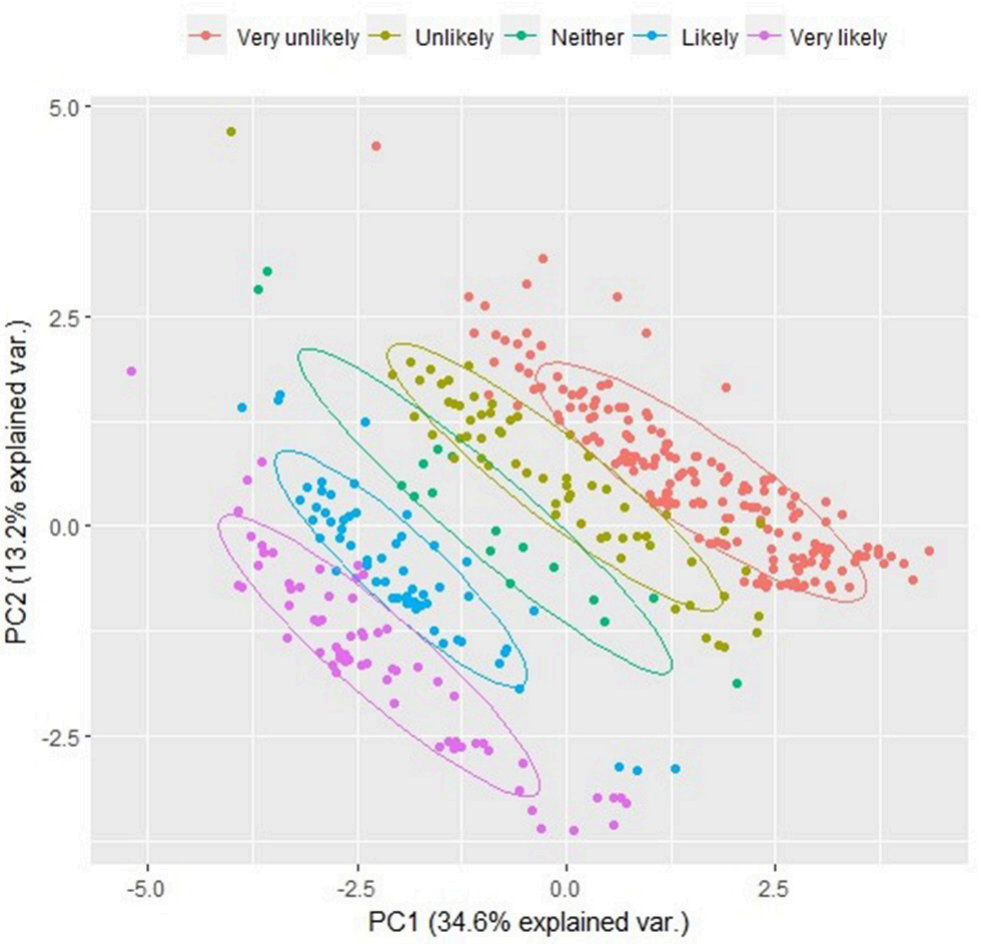
First and second principal components. Colors represent the likelihood of arranging veterinary treatment, which is aligned on both the first and second principal component.

To explore the attitudes, knowledge and perceptions that underpin the likelihood that individuals will arrange neutering for unowned cats, an initial exploratory PCA was used to assess the degree to which different survey items were aligned. This approach reduced the dimensions of the data to principal components, which incorporated the variables that had the highest correlations. The first two principal components explained almost 50% of the variation in the data. Intended behavior was correlated with both PC1 and PC2, allowing identification of key variables that were also correlated with the principal components (Figure 2 and Table 3).

**Table 3:** The latent variables and underlying survey items, alongside the results from a principal component analysis.

**Latent variable/survey item**	**Loadings (1st PC)**	**Loadings (2nd PC)**
**BEHAVIORAL INTENT**		
How likely are you to arrange or take an unneutered cat, which you believe to be unowned, to the vet to be neutered?	0.58	0.73
**KNOWLEDGE OF UNOWNED CATS NEEDS**		
How important do you think it is that unowned cats are provided with neutering?	0.14	
How important do you think it is that unowned cats are provided with treatment?	0.16	
**KNOWLEDGE OF NEUTERING**		
Disagree that related cats won't mate with each other		0.10
Agree that neutering reduces anti-social cat behavior, like wailing and spraying		0.10
**PERCEPTIONS OF UNOWNED CATS**		
Total number of bad points respondents stated	0.23	
**ATTITUDE TOWARD CATS**		
How much do you like or dislike cats?	0.59	0.49
Do you own a cat?	0.13	0.13

*The first and second principal components (PC) are aligned with behavior intent. Variables with loadings >0.10 are shown here. The higher the component loading the more important it is and therefore aligns with behavioral intent*.

PCA is limited in that it is correlative. To incorporate both correlative and causative effects we explored links between interrelated variables using structural equation models (SEMs) using package Lavaan (23) in program R v. 3.4.3 (24). We used a chi-square test, the root mean square error of approximation (RMSEA), and the comparative fit index (CFI) as measures of model fit for the final model, according to the following criteria (25): (1) *P*-values of chi-square tests > 0.05; (2) lower 90% confidence intervals of RMSEA close to 0; and (3) CFIs ≥0.9.

Our starting model explored whether behavioral intent was driven by attitude, perceptions and knowledge of neutering and the needs of unowned cats. Correlations between all drivers were also incorporated in the model. Significance was consequently assessed by examining standard errors and *P*-values associated with each SEM path.

**Figure 3:**
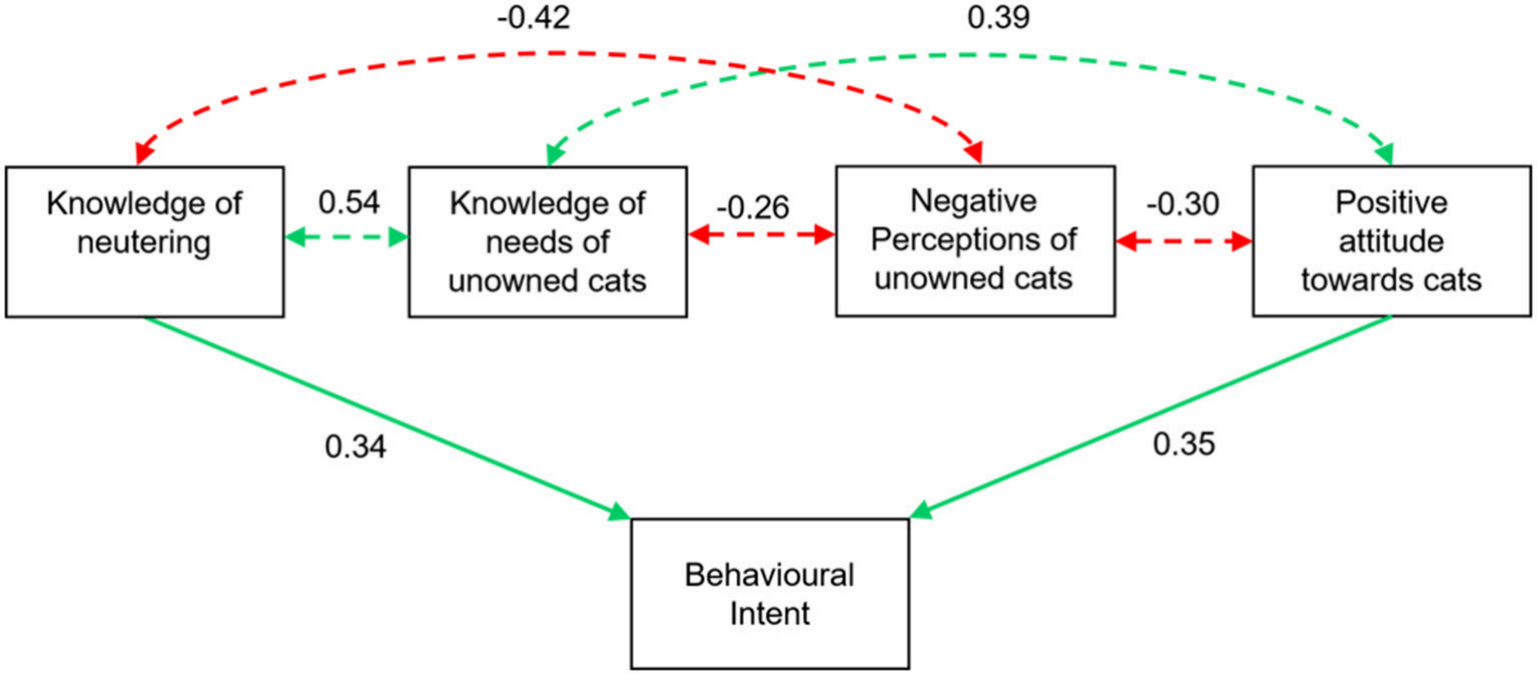
Path diagram used in final structural equation model. See Table 3 for definition of variables. Solid lines indicate significant direct effects and dashed lines indicate significant correlative effects. Green show positive effects and red negative effects.

Our final model indicated that behavioral intent to arrange neutering for an unowned cat was caused by knowledge of neutering and a positive attitude toward cats (Figure 3). However, there were also numerous indirect effects due to correlations between perceptions and knowledge regarding the needs of unowned cats. This final SEM fit the data well (χ2 = *19.11, p* = 0.161; RMSEA = 0; CFI = 0.99).

### TNR campaign and interventions

In the first instance, interventions focussed on targeting the behavior of reporting stray cats to make this the “norm.” The provision of different channels to report cats and a strong community presence both face-to-face and online all helped develop both the capability and opportunity to report unowned cats.

To increase motivations, the findings of the survey indicated that knowledge and attitude toward cats had the strongest influence of behavioral intent. Consequently, this was used to inform umbrella messaging, which highlighted the benefits of having stable, neutered cat populations, and how TNR would be used to achieve this. Additionally, increasing knowledge of neutering within the community, through face-to-face events and online, helped to improve behavioral intent.

Prior to TNR, 3 months of data on the whereabouts of strays were collected identifying hot spots and targeting TNR to specific streets. Actual TNR started in November 2016, and included simultaneous engagement within hot spot areas through door knocking, leafletting and posters, alongside TNR operations. Advice was provided to 1,200 households across 16 hotspot streets, via direct conversations or the posting of an information leaflet. Consequently, the TNR operation itself provided value as both a process and an intervention. As cats underwent TNR the public were concomitantly provided with information as to correct reporting of cats, appropriate food, water, and shelter provision, and how to help with trapping. Therefore, TNR helped to improve the community's sense of agency. Additionally, seeing the benefits of neutered cats through TNR also had to potential to counteract negative perceptions.

### Outcomes

The direct and immediate benefits of community engagement and TNR are the numbers of unowned cats that are taken in for neutering and either returned to the community or rehomed when appropriate. Intelligence regarding unowned cat hotspots was obtained from the 776 household surveys. In addition, a variety of ways to report cats were put in place, including mobile application, face-to-face, and online. This resulted in 124 individual reports of unowned cats within the Bulwell community. This information enabled identification of hotspots and targeted TNR, resulting in 104 unowned cats taken in for neutering, of which 87 were returned and 17 were euthanized on veterinary advice due to poor welfare. In addition, a further 51 unowned cats were fit for rehoming and 7 cats were already microchipped and therefore subsequently reunited with their owners. Further to this, 92 owned cats were neutered as an indirect consequence of the Bulwell Cat Watch campaign specifically.

Long-term benefits of community engagement and TNR will come from behavior change relating to positive neutering practices. Alongside engagement at the permanent hub, between October 2016 and July 2018, the community engagement team attended or ran 29 community events, these included fun days, talks to local community groups including schools and stalls at local community events, with an estimated engagement of over 1,000 individuals. Online engagement via a Facebook group created an active community with ~600 members, averaging 41 posts in 30 days, reported in July 2018.

In addition, 11 people from the Bulwell community are now actively volunteering to aid community cats in various registered roles such as TNR volunteer, social media volunteer, and community project officer volunteer.

Although, further evaluation of long-term community impacts is needed. Initial evaluation surveys of 54 residents undertaken after TNR had started indicated that the majority of people perceived the Cat Watch to be good or very good for Bulwell cats (96%) and also the community generally (90%). Specifically, Cat Watch was perceived to help the unowned cats, provide support to enable people to help stray cats and raise awareness about stray cat numbers.

**“Because it is making us aware to look out for and help stray cats. Didn't know there was so many strays.”**

**“Some people don't know what to do about stray cats or how to get them help or find information to help the stray cats.”**

Also, responses suggest that Bulwell Cat Watch is changing awareness, attitudes and behavior. With most people agreeing or strongly agreeing that they are more concerned about unowned cats, more aware of cat welfare issues in the community and they will do more to help unowned cats.

**“They do an excellent job to improve the stray cat population. Neuter and give care when a cat is reported that needs help. Made community more aware of problems and advise [sic].”“It has given me advice on how to deal with a stray cat.”**

## Discussion

Without continued neutering within the community, TNR work is at risk of failing to make significant progress in urban areas and its long-term impact jeopardized (3). We highlight a framework, whereby TNR operations can be embedded within community engagement. Interventions are therefore able to create a legacy of behavior change that is more likely to continue once TNR operations have ceased. We also demonstrate how statistical modeling approaches can identify the direct and indirect basis for desirable behaviors toward cats and aid tailoring of such interventions. Results from our case study affirm the idea that human cognitive biases, emotions, and behavior toward unowned cats are complex and interrelated.

We found that intended behavior toward cats was primarily driven by attitudes toward cats in general. Perceptions about unowned cats also shaped attitudes, suggesting there are higher order cognitions that strongly influence behavioral intentions. This result is not dissimilar from previous studies (5), which found people are more likely to care about cats if they perceive them positively. The most commonly reported problem created by unowned cats was their perceived dirtiness. This may reflect the importance of the community to individuals and their perceived inability to control their own environment. The intervention tools employed, including communications that highlight the benefits of population control and demonstrating how to help unowned cats, may improve perceptions of unowned cats and increase sense of agency in order to improve the community and its cleanliness. Although the extent to which social norms influence attitudes and perceptions was not included in the SEM illustrated in this paper, the perception to be “doing the right thing” is likely to help develop positive feelings toward helping cats. Consequently, interventions that increased visibility of positive behavior toward cats through a strong community presence and Facebook groups, provided another means of modeling behavior by residents. In particular, the Facebook group network that formed as part of this project, and the people within it, have the ability to share information and influence each other. As co-members of the same group people start to build relationships with each other, at a level and scale not possible solely through a TNR team.

Respondents knowledge was the second predictor of intentions. More than half of respondents were either unsure or agreed that female cats should be allowed to have a litter prior to being neutered and that related cats would not mate with each other. This result is consistent with previous studies that identified poor owner knowledge of feline reproduction (26). Additionally, knowledge regarding the needs of unowned cats was also indirectly linked to intended behavior toward them. Provision of educational material alongside demonstration of desirable behavior toward cats will also be a valuable intervention to reduce the number of unwanted litters. Preliminary outcomes from such interventions are positive with high neutering rates and positive community feedback, however going forward more in-depth evaluation will be required to explore whether both intentions and actual behavior to help unowned cats has increased.

Early engagement with the community prior to and during TNR operations had the additional operational benefit of identifying perceived areas of high unowned cat density. This is valuable as unowned densities have been found to vary dramatically even across a short distance (27). Additionally, such engagement can build trust and improve communication with cat caretakers, enabling access to previously unidentified colonies. This included those located behind homes and businesses, access to which has been identified as a logistical constraint in previous TNR programs (27). Our case study highlighted the value of different communication channels for reporting unowned cats (e.g., online, phone application, and face-to-face), alongside the initial intelligence from the survey. This intelligence resulted in targeted engagement and high rates of TNR in areas where it was most needed.

As our case study shows, the combination of survey instruments with modeling approaches can inform how best to approach community engagement and interventions. The hierarchical relationships among variables would have been missed if a multiple linear regressions were used. The modeling framework presented here is easily extended to incorporate a range of behavioral concepts and provides the basis to explore different behavioral hypothesizes. The adaptability of this approach is desirable as barriers and motivations will vary across communities and depend on the sociodemographic context (3), therefore there is much to be gained from the insight that community-level empirical data can provide. However, we note the quantitative nature of this modeling approach limits its applicability in situations where qualitative data are collected. For example, focus groups, although not discussed here, are an important source of knowledge to determine intervention approaches.

In highly deprived areas, the barriers to unowned cat management will also encompass broader community problems. Going forward, this community engagement could spark wider collaborations with other stakeholders and human agencies, such as housing authorities and foodbanks. Providing interventions that work together and address community problems will help empower individuals encouraging positive behavior. For example, offering social support has shown to be important for behavior change in other contexts (28–31), but is currently missing from traditional TNR approaches.

## Conclusion

We have highlighted the importance of accounting for anthropogenic factors when determining appropriate interventions to ensure the long-term benefit of TNR campaigns. Our case study revealed the drivers of behavioral intention go far beyond a lack of awareness alone and that attitudes, perceptions and knowledge are all significant drivers. Studies that fail to account for specific barriers around helping unowned cats within a community may not effectively increase the capacity for people to help unowned cat populations and prevent cat overpopulation more generally. This study adds to the increasing understanding that targeted interventions are necessary for behavior change. We therefore recommend further consideration of the social context within which TNR is often implemented and ultimately application of similar approaches across other urban areas around the world.

## Ethics statement

All questionnaires were optional, and respondents were advised they did not need to take part and could withdraw involvement at any point. This study was carried out in accordance with the recommendations of University of Bristol's Ethics Policy and Procedure. The protocol was approved by University of Bristol Faculty of Health Science Research Ethics Committee approval number 38661.

## Author contributions

JM coded the models, ran the analysis and wrote the paper. JC motivated the research, manages the Cat Watch project and commented on drafts. MF provided constructive input into the development of the project and commented on drafts.

### Conflict of interest statement

The authors declare that the research was conducted in the absence of any commercial or financial relationships that could be construed as a potential conflict of interest.
