# Supplementary material for: Integrating Trap-Neuter-Return Campaigns Into a Social Framework: Developing Long-Term Positive Behavior Change Toward Unowned Cats in Urban Areas
Source: Front Vet Sci. 2018 Oct 24;5:258. doi: 10.3389/fvets.2018.00258 (PMC6207997; doi:10.3389/fvets.2018.00258)
Supplement: Supplementary file 1 [file Table_1.DOCX]

# Questions from Cat Watch survey

q1. In general, how much would you say you like or dislike cats?

❑ 1. Like a lot

❑ 2. Like a little

❑ 3. Indifferent

❑ 4. Dislike a little

❑ 5. Dislike a lot

q2. Approximately how many cats do you think there are around where you live? [If prompted – this can include ‘pets’ or ‘owned cats’]

❑ 1. None

❑ 2. 1-2

❑ 3. 3-4

❑ 4. 5-9

❑ 5. 10 or more

❑ 6. Don’t know

q3. Using your best guess, how many of these cats do you think are household pets?

❑ 1. None

❑ 2. 1-2

❑ 3. 3-4

❑ 4. 5-9

❑ 5. 10 or more

❑ 6. Don't know

q4. Now thinking about the cats around here that are not household pets. Using your best guess, how many of these unowned cats do you think are fed or looked after by local people?

❑ 1. None

❑ 2. 1-2

❑ 3. 3-4

❑ 4. 5-9

❑ 5. 10 or more

❑ 6. Don’t know

q5. Do you think having unowned cats in the local area, is (or would be) good or bad for the community on the whole?

❑ 1. Good

❑ 2. Neither good nor bad

❑ 3. Bad

q6. What, if any, do you think are the good points of having unowned cats around where you live? (Unprompted response, tick all that apply)

❑ 1. Vermin control

❑ 2. Encourages good community connections

❑ 3. Good for children

❑ 4. Like seeing them around

❑ 5. Like seeing the kittens

❑ 6. None

❑ 7. I have never thought about it

❑ 8. Other

Please specify other:

q7. What, if any, do you think are the bad points of having unowned cats around where you live? (Unprompted response, tick all that apply)

❑ 1.Fight with pet cats

❑ 2. Breed with pet cats

❑ 3. Make a noise

❑ 4. Smell

❑ 5. Dirty

❑ 6. Makes the area look run down

❑ 7. Sets off security lights

❑ 8. None

❑ 9. I have never thought about it

❑ 10. Other

Please specify other:

q8. How important do you think it is that unowned cats are provided with…..?

|  | 1. Very important | 2. Quite important | 3. No opinion | 4. Not very important | 5. Not at all important | 6. Don’t know |
| --- | --- | --- | --- | --- | --- | --- |
| 1. Food | ❑ | ❑ | ❑ | ❑ | ❑ | ❑ |
| 2. Water or milk | ❑ | ❑ | ❑ | ❑ | ❑ | ❑ |
| 3. Shelter | ❑ | ❑ | ❑ | ❑ | ❑ | ❑ |
| 4. Vaccinations | ❑ | ❑ | ❑ | ❑ | ❑ | ❑ |
| 5. Vet treatment when they are sick or injured | ❑ | ❑ | ❑ | ❑ | ❑ | ❑ |
| 6. Neutering (e.g. access to neutering programmes run by charities) | ❑ | ❑ | ❑ | ❑ | ❑ | ❑ |

q9. Which if any of the following do you provide or organise, currently or in the past year, for unowned cats around where you live?

|  | 1. Yes | 2. No | 3. Don’t know |
| --- | --- | --- | --- |
| Food | ❑ | ❑ | ❑ |
| Water or milk | ❑ | ❑ | ❑ |
| Shelter | ❑ | ❑ | ❑ |
| Vaccinations | ❑ | ❑ | ❑ |
| Vet treatment when they are sick or injured | ❑ | ❑ | ❑ |
| Neutering (e.g. access to neutering programmes run by charities) | ❑ | ❑ | ❑ |

q10. And which, if any, of the following do you think are provided or organised by others, currently or in the past year, for unowned cats around where you live?

|  | 1. Yes | 2. No | 3. Don’t know |
| --- | --- | --- | --- |
| Food | ❑ | ❑ | ❑ |
| Water or milk | ❑ | ❑ | ❑ |
| Shelter | ❑ | ❑ | ❑ |
| Vaccinations | ❑ | ❑ | ❑ |
| Vet treatment when they are sick or injured | ❑ | ❑ | ❑ |
| Neutering (e.g. access to neutering programmes run by charities) | ❑ | ❑ | ❑ |
| Never thought about it | ❑ | ❑ | ❑ |

q11. How likely are you to arrange or take an unneutered cat, which you believe to be unowned, to the vet to be neutered?

❑ 1.Very likely

❑ 2. Likely

❑ 3. Neither

❑ 4. Unlikely

❑ 5. Very unlikely

❑ 6. Don’t know

q12. What, if anything, would stop you from taking a community cat to a vet to be neutered?

❑ 1. Can’t catch

❑ 2. Cost

❑ 3. The time/hassle for me

❑ 4. Don’t want to stress the cat

❑ 5. Didn’t think it was my responsibility

❑ 6. Not interested in cats

❑ 7. Might be somebody else’s cat

❑ 8. Hasn’t crossed my mind

❑ 9. Don’t agree with neutering

❑ 10. Risk of being injured

❑ 11. Don’t think it’s important

❑ 12. Don’t know

❑ 13. Never thought about it

❑ 14. Other

Please specify other:

q13. Do you know that animal charities can help to get unowned cats neutered?

❑ 1. Yes

❑ 2. No

❑ 3. Don’t know

q14. Compared to healthy pet cats (cats that are not too skinny and have clean shiny coats of fur) how would you describe the overall health of the unowned cats around where you live?

❑ 1. Very good – no concerns

❑ 2. Quite good – 1 or 2 concerns

❑ 3. Quite poor – a few concerns

❑ 4. Very poor – many concerns

❑ 5. Don’t know / no opinion

q15. Who do you think should be responsible for looking after unowned cats in your area?

❑ 1. Everyone in the community

❑ 2. Those in the community with an interest in the cats

❑ 3. The council / housing association

❑ 4. Animal charities

❑ 5. None of these

❑ 6. Don’t know

❑ 7. Other

Please specify other:

q16. What is the youngest age you think a female cat can become pregnant?

q17. I’m going to read you a series of statements and will ask you whether or not you agree or disagree with each statement. There are no right or wrong answers – just tell me what you believe.

|  | 1. Agree | 2. Neither agree nor disagree | 3. Disagree | 4. Don’t know |
| --- | --- | --- | --- | --- |
| Neutering is a good way to reduce the number of cats | ❑ | ❑ | ❑ | ❑ |
| Neutering is cruel | ❑ | ❑ | ❑ | ❑ |
| Neutering reduces anti-social cat behaviour, like wailing and spraying | ❑ | ❑ | ❑ | ❑ |
| Female cats should be allowed to have kittens before being neutered | ❑ | ❑ | ❑ | ❑ |
| Related cats won’t mate with the each other | ❑ | ❑ | ❑ | ❑ |

q18. If any, how many pet cats do you own?

q19. Are all of your cats neutered?

❑ 1. Yes

❑ 2. No

❑ 3. Don’t know

q20. What are the ages of the cats that haven’t been neutered?
